# Management of Hypercholesterolemia in Patients with Coronary Artery Disease: A Glimpse into the Future

**DOI:** 10.3390/jcm13237420

**Published:** 2024-12-05

**Authors:** Alessandro Sciahbasi, Paola Russo, Michela Zuccanti, Laura Chiorazzo, Francesco Maria Castelli, Antonino Granatelli

**Affiliations:** 1 Interventional Cardiology, Sandro Pertini Hospital, 00157 Rome, Italy; antonino.granatelli@aslroma2.it; 2 Cardiology, Sant’Andrea Hospital, 00189 Rome, Italy; russo.2018832@studenti.uniroma1.it (P.R.); michela.zuccanti@uniroma1.it (M.Z.); 3 Cardiology Unit, University of L’Aquila, 67100 L’Aquila, Italy; laura.chiorazzo@graduate.univaq.it; 4 Cardiology Unit, University of Pisa, 56126 Pisa, Italy; castellifrancesco98@gmail.com

**Keywords:** hypercholesterolemia, statin, evolocumab, alirocumab, inclisiran, ezetimibe, bempedoic acid, PCSK9i, Lipoprotein (a)

## Abstract

Cardio-cerebral vascular diseases due to atherosclerosis are still the leading cause of death worldwide. Low-density lipoprotein cholesterol (LDL-C) and apolipoprotein B have been identified as the primary factors responsible for the atherosclerotic process, with a causal effect. Many drugs aimed at reducing LDL-C levels are already on the market, acting in different ways in terms of mechanism of action, efficacy, and safety. Moreover, new lipid-lowering agents and new technologies in the fields of gene editing and immunotherapy are currently under investigation. A more recent biomarker associated with an increased risk of plaque generation, progression, and subsequent ASCVD is the lipoprotein (a) and, in the next few years, it will be the new target of pharmacological therapy. The aim of this review is to present the landscape of therapies already approved to reduce LDL-C levels, evaluating their efficacy, tolerability, and indications. Moreover, we take a glimpse into the future to evaluate experimental novel therapies to lower LDL-C levels that will be approved in the next few years or are under clinical evaluation.

## 1. Introduction

Cardio-cerebral vascular diseases, mainly ischemic heart disease and stroke due to atherosclerosis, are the leading cause of mortality and disability worldwide, responsible for 18 million deaths each year and accounting for 32% of all global deaths in 2019 [1]. Different cardio-metabolic, behavioral, environmental, and social risk factors are associated with the atherosclerotic process [2] and among them, dyslipidemia is a crucial factor. In particular, low-density lipoprotein cholesterol (LDL-C) has been identified as a key initiating event in atherogenesis with a causal and cumulative effect [3]. In this context, lipid-lowering therapies are able to stop the atherosclerotic process, stabilize the vascular plaques, and reduce cardiovascular risk. Studies aimed at lowering LDL-C levels have shown a 40–50% reduction in events for every 38 mg/dL drop of LDL-C [4,5,6].

**Figure 1:**
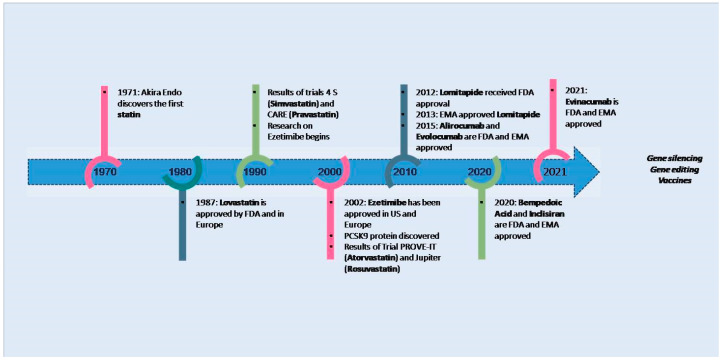
Major discoveries in treatment of hypercholesterolemia. Since the discovery of statin in 1971 by Akira Endo, multiple improvements in low-density lipoprotein cholesterol have been obtained, and in the near future we are waiting for primary results on studies with gene editing and vaccines. EMA: European Medical Agency; FDA: Food and Drug Administration; PCSK9: Proprotein convertase subtilisin/kexin type 9.

Statins still represent the cornerstone of therapy in the management of dyslipidemia due to their low cost, limited side effects, and elevated efficacy, as confirmed in clinical studies [7]. However, statin monotherapy, even at high doses, often cannot reach the guideline-recommended goals for LDL-C and is burdened by side effects, inter-individual variability, and poor patient adherence. Therefore, multiple adjunctive drugs have been tested to improve the efficacy of statins and reduce side effects [8]. In this regard, several placebo-controlled clinical trials have demonstrated that the addition of ezetimibe and anti-proprotein convertase subtilisin/kexin type 9 (PCSK9) monoclonal antibodies to statin treatment produces more favorable results in terms of achieving LDL-C target levels and in terms of the improvement of all lipid parameters considered (Table 1). In addition, new lipid-lowering agents, such as inclisiran and bempedoic acid, have received approval and new technologies in the field of gene editing and immunotherapy, like vaccines, are currently being investigated (Figure 1).

The aim of this review is to display the landscape of current therapies for the treatment of hypercholesterolemia, discuss the optimal treatment of lipid control, and offer a glimpse into the future of novel therapies.

## 2. Cholesterol as a Causative Agent in the Atherosclerotic Process

Cholesterol plays several fundamental functions in our body, such as the formation of cell membranes and the production of hormones, but it is also associated with the development of atherosclerosis, as observed since 1913 by the pathologist Nikolai Anitschkow who stated: “There is no atherosclerosis without cholesterol” [9]. In subsequent years, the role of cholesterol (in particular LDL-C, but also very low-density lipoproteins and their remnants, intermediate-density lipoproteins, and smaller triglyceride-rich lipoproteins) has been fully elucidated and now cholesterol is recognized not only as a risk factor for atherosclerosis but more importantly as a causative agent responsible for atherosclerotic cardiovascular disease (ASCVD) [3].

**Figure 2:**
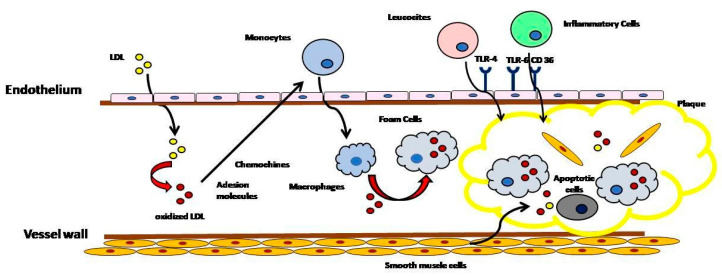
Schematic representation of the atherosclerotic process. LDL-C interacts with the proteoglycan matrix and accumulates in the arterial wall and is susceptible to oxidation and modification to a toxic form, generating oxidized LDL-C (oxLDL-C). OxLDL-C activates endothelial cells to produce adhesion molecules and chemokines to recruit monocytes into the arterial wall, which, after differentiation into macrophages, internalize oxLDL-C through the scavenger receptor and become foam cells. These cells induce the production of chemokines and the expression of receptors (TLR-4, TLR-6, and CD36) on endothelial cells.

Fatty streaks are the first observable lesions in the atherosclerotic process due to infiltrates of LDL-C into the sub-intima through a transcytotic process [10]. Progressively, LDL-C interacts with the proteoglycan matrix that promotes retention in the subintima mediated by the interaction between arginine and lysine contained in apo B100 (positively charged) and the sulfate and carboxylic acid groups contained in arterial wall proteoglycans [11]. LDL-C, accumulated in the arterial wall, creates plaque and is susceptible to oxidation and modification to a toxic form mediated by enzymatic and non-enzymatic mechanisms [12,13], generating oxidized LDL-C (oxLDL-C). OxLDL-C activates endothelial cells to produce adhesion molecules and chemokines to recruit monocytes into the arterial wall [14], which, after differentiation into macrophages, internalize oxLDL-C through the scavenger receptor and become foam cells [15]. These cells induce the production of chemokines (CXCL1) and the expression of receptors (TLR-4, TLR-6, and CD36) on endothelial cells [16], which support the inflammatory process and facilitate the recruitment of mast cells, CD8+ T cells, and CD4+ cells, leading to plaque progression [17,18]. As a final result, apoptotic cells accumulate in the sub-intimal space and undergo a necrotic process, resulting in a necrotic core that leads to a plaque that is susceptible to rupture (Figure 2).

## 3. LDL-C Therapeutic Target Levels

Multiple studies have shown that lowering LDL-C levels is directly correlated to a reduction in cardiovascular events [4,5,6], and the absolute benefit relies not only on the amount of LDL-C reduction but even more importantly on the absolute basal risk of ASCVD of the patient: the higher the risk, the higher the benefit. This means that the target therapeutic levels to be reached rely on the individual cardiovascular risk and the intensity of treatment ranges from lifestyle changes to aggressive drug treatment [7] according to baseline LDL-C levels.

**Figure 3:**
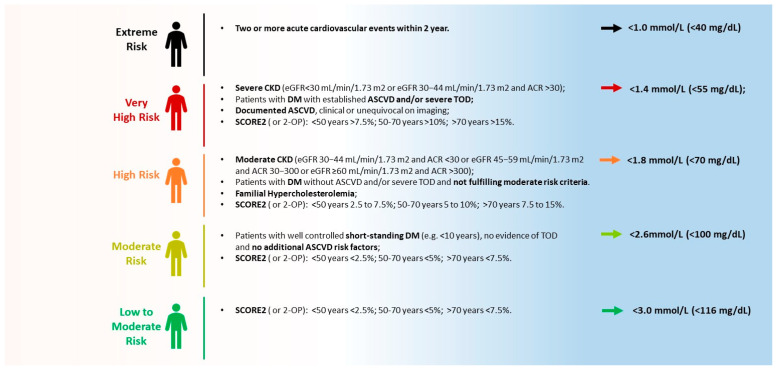
Latest European Society of Cardiology Guidelines. According to guidelines, patients are classified into 5 different risk score classes with different therapeutic low-density lipoprotein cholesterol targets. ACR: albumin to creatinine ratio; ASCVD: atherosclerotic cardiovascular disease; CKD: chronic kidney disease; DM: diabetes mellitus; eGFR: estimated glomerular filtration rate; OP-2: Older Persons-2; SCORE-2: Systematic Coronary Risk Estimation; TOD: target organ damage.

According to the latest ESC Guidelines on cardiovascular disease prevention published in 2021 [19], people with documented ASCVD, type 1 or type 2 diabetes, very high levels of individual risk factors (such as familial hypercholesterolemia), or chronic kidney disease are generally at high or very high total cardiovascular risk, and LDL-C levels should be lower than 55 mg/dL (1.4 mmol/L) for those at very high risk and lower than 70 mg/dL (1.8 mmol/L) for patients at high risk. Moreover, if baseline LDL-C levels are below these thresholds, then a 50% reduction in baseline LDL-C levels should be obtained (Figure 3). Patients who experience a second vascular event within 2 years are considered at extreme risk and an LDL-C goal of <40 mg/dL (<1.0 mmol/L) may be considered (Figure 3).

For apparently healthy individuals (primary prevention), the use of a risk estimation system such as the SCORE2, which estimates the 10-year cumulative risk of a first fatal and non-fatal CVD event for patients aged 40–69 years, is recommended in order to implement the most accurate preventive strategies for the subject. According to the SCORE 2 results, individuals can be classified as having low, moderate, or high cardiovascular risk and the LDL-C target levels are set accordingly (Figure 2). There are also dedicated score charts for some subgroups of patients: in apparently healthy people aged ≥70 years, the use of SCORE 2-OP is suggested, whereas in patients with diabetes, the SCORE2-Diabetes should be employed [20]. In all patients, a reclassification of asymptomatic low- or moderate-risk patients should be considered in the case of the detection of atherosclerotic disease on arterial ultrasound of the peripheral vessels (carotid/femoral) or on the basis of the Agatson coronary calcium score [21].

## 4. Lipid-Lowering Strategies: From Consolidated Approaches to Innovative Therapies

LDL-C remains the main target of lipid-lowering treatment, and the intensity and rapidity of therapy should be tailored according to the basal LDL-C level and the individual ASCVD risk. For example, in primary prevention, a staged strategy is recommended comprising lifestyle interventions (class I), followed by high-intensity statin therapy at the maximum tolerated dose (class I), then the addition of ezetimibe (class I), and finally PCSK9 inhibitors (PCSK9i) if the LDL-C target is not attained [19]. Differently, a combination therapy with statin and ezetimibe or a PCSK9 inhibitor as an initial strategy for the secondary prevention of cardiovascular events is strongly recommended (class I), bearing in mind the treatment average reduction in LDL-C that can be obtained with each different therapy. For example, LDL reduction ranges from almost 30% for moderate-intensity statins to more than 80% for the combination of PCSK9 inhibitor plus high-intensity statin plus ezetimibe (Table 1).

The evidence of efficacy and safety of consolidated therapies and new emerging approaches is now presented in order to display the large armamentarium available to control cholesterol levels.

### 4.1. Statins

**Figure 4:**
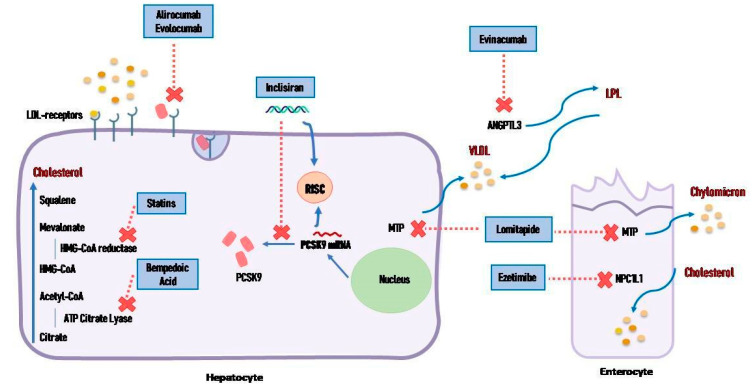
Mechanism of action of drugs commercially available. Statins block the 3-hydroxy-3-methyl-glutaryl-coenzyme A (HMG-CoA) reductase, the enzyme that converts HMG-CoA into mevalonic acid. Ezetimibe inhibits the Niemann-Pick C1-like protein 1 at the level of the brush border of the intestine, reducing absorption of dietary and biliary cholesterol and its incorporation into chylomicrons. Bempedoic acid inhibits an ATP citrate lyase responsible for cholesterol synthesis in the same enzymatic cascade target of statins, but upstream of HMG-CoA reductase. Monoclonal antibodies are directed against circulating proprotein convertase subtilisin/kexin type 9. Small interfering RNAs are short RNA molecules that interfere with the production of different proteins through a “carrier” (viral or non-viral vectors), forming the RNA-induced silencing complex that binds to the target complementary mRNA, cleaving it. Lomitapide is an oral selective inhibitor of the microsomial triglyceride transfer protein, decreasing the production of triglycerides, chylomicrons and very low-density lipoproteins. Evinacumab is a monoclonal antibody blocking ANGPTL-3 that inhibits lipoprotein lipase, responsible for hydrolysis of triglycerides content of very low-density lipoproteins. ANGPTL: Angiopoietin-like proteins, HMG-CoA: hydroxy-3-methyl-glutaryl-coenzyme A, LDL: low-density lipoprotein, LPL: lipoprotein lipase, MTP, microsomial triglyceride transfer protein, NPC1L1: Niemann-Pick C1-like protein 1, PCSK9: proprotein convertase subtilisin/kexin type 9.

Statins, for their documented efficacy, low cost, and limited side effects, remain the first-line treatment for lipid control [19]. They block the 3-hydroxy-3-methyl-glutaryl-coenzyme A (HMG-CoA) reductase, the enzyme that converts HMG-CoA into mevalonic acid, a cholesterol precursor, followed by up-regulation of the low-density lipoprotein receptor (LDL-R) at the surface of the hepatocytes, which in turn results in increased uptake of LDL [22] (Figure 4). The effect of statins on LDL-C circulating levels depends on the dose, type, and individual response. In particular, according to the intensity, statins are classified into three categories: high-intensity, moderate-intensity, and low-intensity. High-intensity statin therapy typically lowers LDL-C levels by ≥50%, moderate-intensity statin therapy by 30% to 49%, and low-intensity statin therapy by <30% [23].

Statin therapy is generally well tolerated, even at high doses, and the most clinically relevant adverse effect is statin-induced myopathy, documented in 10–15% of statin users, which is often a common cause of therapy discontinuation [24]. Furthermore, statins have been associated with an increased risk of new-onset diabetes mellitus. Previous observational studies and meta-analyses showed that the risk is higher in the elderly, in the presence of other risk factors for diabetes, and when using high-intensity statins [25].

The use of statins has been associated with a significantly better cardiovascular outcome compared to a placebo in primary as well as secondary prevention trials. The first study that showed a significant reduction in the rate of acute myocardial infarction in patients with elevated LDL-C levels but without coronary artery disease with pravastatin compared to a placebo was the West of Scotland Coronary Prevention Study Group (WOSCOPS) trial [26]. These results were confirmed in the AirForce/Texas Coronary Atherosclerosis Prevention Study (AF-CAPS/TexCAPS) [27] with lovastatin, in the Anglo-Scandinavian Cardiac Outcomes Trial-Lipid Lowering Arm (ASCOT-LLA) [28] with atorvastatin, and in the Justification for the use of statins in prevention (JUPITER) trial [29] with rosuvastatin. Globally, the number needed to treat (NNT) to avoid a cardiovascular event ranged from 83 patients to 169 per year according to baseline LDL-C levels (the higher the LDL-C levels, the greater the benefit). Similarly, in secondary prevention, statin use was associated with a significant reduction in cardiovascular events compared to a placebo, and this reduction was inversely correlated with the baseline cardiovascular risk and LDL-C levels of the patients enrolled in the studies. The effect was independent of the statin employed: simvastatin in the Scandinavian Simvastatin Survival Study (4S) trial [30], pravastatin in the Cholesterol and Recurrent Events (CARE) trial [31], or atorvastatin in the Pravastatin or Atorvastatin Evaluation and Infection Therapy (PROVE-IT) trial [32]. According to the higher baseline risk of the patients included in secondary prevention trials, the NNT was lower compared to primary prevention trials, accounting for only 30 patients in the 4S trial [30]. The results of a meta-analysis by Baigent et al. clarified the proportionality of the relationship between LDL-C and cardiovascular events, highlighting that for each 1 mmol/l or 38 mg/dL reduction in LDL values, there is a 12% reduction in mortality, essentially related to a reduction in mortality from coronary causes, and a 21% reduction in major cardiovascular events [4].

In patients with advanced chronic kidney disease, the cardioprotective role of statin appeared reduced in the Rosuvastatin in Subjects on Regular Hemodialysis: An Assessment of Survival and Cardiovascular Events (AURORA) Trial [33]. However, different observational studies showed that chronic treatment with statin in patients with moderate renal impairment and presenting with acute coronary syndromes was associated with better in-hospital and long-term outcomes compared to a placebo [34,35].

**Table 1:** Expected reduction of LDL-c levels compared to placebo.

Drug Class	Expected Proportional LDL-C Lowering Compared with Placebo
Moderate-intensity statin [23]	30%
High-intensity statin [23]	50%
Ezetimibe [36]	20%
PCSK9 antibody [37]	60%
PCSK9 siRNA [38]	50%
Bempedoicacid [39]	15–25%
Combination therapy high-intensity statin plus Ezetimibe [40]	65%
High-intensity statin plus PCSK9 antibody [37]	75%
High-intensity statin plus Ezetimibe plus PCSK9 antibody [37]	85%
Bempedoic acid plus Ezetimibe [41]	35%
Evinacumab (Angiopoietin-like protein-3 inhibitor) [42]	50%
Lomitapide [43]	50%
Gene editing [44]	69%

LDL-C L: low-density lipoprotein cholesterol, PCSK9: Proprotein convertase subtilisin/kexin type 9.

### 4.2. Ezetimibe

Ezetimibe is an azetidine derivative that inhibits the Niemann-Pick C1-like protein 1 (NPC1L1) at the level of the brush border of the intestine, reducing the absorption of dietary and biliary cholesterol and its incorporation into chylomicrons (Figure 4). Consequently, the intrahepatic cholesterol pool decreases and LDL-R expression is up-regulated, increasing the clearance of ApoB100-containing lipoproteins from plasma [45]. The drug is well tolerated, without the muscle side effects observed during statin therapy.

Different studies showed that ezetimibe as monotherapy at a dose of 10 mg/day reduces LDL-C in hypercholesterolemic patients by 18%, although with relatively high inter-individual variation [36]. However, when ezetimibe is used as an adjuvant therapy with statins, it results in a supplementary 24% reduction in LDL-C compared to statin monotherapy [40,46]. In particular, in the Improved Reduction of Outcomes: Vytorin Efficacy International Trial (IMPROVE-IT) the association of ezetimibe (10 mg) with simvastatin (40 mg) resulted in incremental lowering of LDL cholesterol levels and improved cardiovascular outcomes compared to simvastatin alone [46].

### 4.3. Proprotein Convertase Subtilisin/Kexin Type 9 Inhibitors

PCSK9 is a functional protein released by the liver that acts in lipid homeostasis like an LDL receptor-binding protein. It induces lysosomal catabolism of LDL-receptor (LDL-R) and a consequent increase in plasma LDL concentrations [37]. The use of monoclonal antibodies (mAbs) directed against circulating PCSK9 allows the reduction of plasma level of PCSK9, which in turn is not available to bind LDL-R, increasing the receptor expression on the cell surface and the reduction of circulating LDL-C levels [37] (Figure 4).

Currently, two fully human mAbs against PCSK9 (alirocumab and evolocumab) are approved and are on the market. Evolocumab and alirocumab can be administered alone or in combination with other lipid-lowering therapies. When combined with high-intensity or maximally tolerated statins, alirocumab and evolocumab reduce LDL-C levels 46-73% more than a placebo [37] and 30% more than ezetimibe [47]. The effect of evolocumab and alirocumab is not confined only to LDL-C levels: they can reduce triglyceride levels, increase high-density lipoprotein cholesterol (HDL-C) and apolipoprotein A-I (ApoA-I) levels [48], and reduce Lp (a) levels by approximately 30–40% [49]. The reduction of LDL-C with PCSK9 inhibitors is also associated with a reduction in major cardiovascular events (composite endpoint of cardiovascular death, myocardial infarction, stroke, and revascularization) by approximately 15–20% in patients with atherosclerotic disease and LDL-C levels >70 mg/dL when added to statin, as observed in the Further Cardiovascular Outcomes Research with PCSK9 Inhibition in Subjects with Elevated Risk (FOURIER) trial [50] and in the Randomized, Double-Blind, Placebo-Controlled, Parallel-Group Study to Evaluate the Effect of Alirocumab on the Occurrence of Cardiovascular Events in Patients Who Have Recently Experienced an Acute Coronary Syndrome (ODYSSEY OUTCOMES) trials [51]. In particular, if the treatment with alirocumab is maintained for ≥3 years, a significant reduction in all-cause mortality of 22% compared to a placebo was observed, and this benefit was most pronounced in patients with baseline C-LDL levels ≥100 mg/dL [52].

A new PCSK9 inhibitor tested in patients with familial and non-familial hypercholesterolemia is Tafolecimab, a fully human monoclonal antibody directed against PCSK9. Phase 1 and phase 3 studies showed a good efficacy and safety in the Chinese population for the 450 mg dose of Tafolecimab every 4 weeks as compared to a placebo, with a significant decrease in LDL-C levels from baseline to week 12 of more than 60% [53,54].

PCSK9 inhibitors are well tolerated: the only side effects reported are itching at the injection site and flu symptoms [55]. Some studies have suggested the possibility of neurocognitive effects [56]; however, the Evaluating PCSK9 Binding Antibody Influence on Cognitive Health in High Cardiovascular Risk Subjects study (EBBINGHAUS) [57], which was specifically designed to detect changes in neurocognitive functions, has refuted this possibility.

An important advantage of PCSK9 inhibitors therapy is the convenient dose frequency (monthly or bimonthly), which enhances patient adherence, but nowadays the major limit to their implementation in clinical practice is undoubtedly still represented by the high cost. In order to overcome the issue of cost and, in some cases, the patient’s reluctance to injectable therapies, clinical research has been pushed in the search for oral PCSK9 inhibitors. NYX-PCSK9 is an orally bioavailable small-molecule inhibitor of PCSK9 that has been effectively tested in mice [58]. It demonstrated a dose-dependent decrease in plasma total cholesterol of up to 57%, which reached 65% when this drug was added to atorvastatin. MK-0616 is another orally available macrocyclic peptide, renally excreted, that blocks PCSK9. In a phase 2b randomized trial including 375 patients at ASCVD risk, MK-0616 was associated with a significant reduction in LDL-C up to 60% compared to a placebo at 8-week follow-up [59]. The rate of adverse events was similar to a placebo and further development of the drug is ongoing.

### 4.4. Bempedoic Acid

Bempedoic acid is a small molecule that inhibits an ATP citrate lyase responsible for cholesterol synthesis in the same enzymatic cascade target as statins, but upstream of HMG-CoA reductase. The result is a reduction of LDL-C and an upregulation of the expression of LDL-R in the liver (Figure 4). Bempedoic acid is a prodrug activated by very long-chain acyl-CoA synthetase-1, located in the liver [60], and an important advantage is that skeletal muscles do not present the activating enzyme, so adverse effects on muscles are potentially reduced (differently from those of statins). It is administered orally in a fixed dose of 180 mg and it is also available in combination with ezetimibe 10 mg.

The first evidence of the effect of bempedoic acid came from the Cholesterol Lowering via Bempedoic Acid, an ACL-inhibiting Regimen (CLEAR OUTCOMES) trial that enrolled statin-intolerant patients. In this trial, which enrolled 13,970 patients, bempedoic acid was associated with a reduction in LDL-C levels of 21% and a significant reduction of the major adverse cardiovascular events of 13% compared to a placebo [39]. Subsequently, in a randomized phase 3 trial enrolling patients at high cardiovascular risk with elevated LDL-C levels, a fixed dose of bempedoic acid and ezetimibe on top of statin or other therapies, significantly reduced LDL-C levels (36% reduction) compared to a placebo [41]. Unfortunately, there are no outcome data for the use of bempedoic acid on top of statin therapy.

The drug is well tolerated, without muscle symptoms or alteration of glucose metabolism (new onset of worsening diabetes mellitus), as observed with statins. The most important adverse effects documented were the increase in acid uric levels and gout (due to a reduction in the tubular excretion of uric acid), cholelithiasis, and elevation of serum creatinine and hepatic enzyme levels. All these side effects are reversible once the drug is suspended [39,41].

### 4.5. Cholesteryl Ester Transfer Protein Inhibitors

Cholesteryl Ester Transfer Protein (CEPT) is a plasma glycoprotein involved in the metabolism of HDL, transferring cholesteryl esters to the apo B-containing glycoproteins from HDL [61]. By blocking CEPT, increased levels of HDL-C have been observed through an increase in cholesterol esters.

In 1990, four Japanese families with CEPT genetic deficiency due to heterozygous and homozygous CEPT mutations were described, showing increased HDL levels and a longer life expectancy [62]. This discovery led to the hypothesis of a protective effect of CETP deficiency, sparking interest in the pharmacological inhibition of CETP. Various CEPT inhibitor drugs have been tested: Dalcetrapib [63] and Evacetrapib [64] showed no significant differences in cardiovascular events compared to a placebo despite a significant increase in HDL-C levels, whereas Torcetrapib was associated with a concerning increase in all-cause mortality due to infections despite an improved cardiovascular outcome and higher HDL-C levels compared to a placebo [65]. Only Anacetrapib showed a significant reduction in cardiovascular events compared to a placebo without significant side effects [66], but the difference was of minimal magnitude and its development and commercialization were halted. Recently, a new CEPT inhibitor tested in clinical practice was Obicetrapib. In a phase 2 dose-finding trial, Obicetrapib demonstrated significant reductions in LDL-C (45%), ApoB (29.8%), non-HDL-C (44%), and Lp (a) (56.5%) with an increase in HDL-C (165%) and Apo A1 (47.8%) levels when added to high-intensity statin therapy and ezetimibe [67]. Thanks to these preliminary studies, Obicetrapib appears to be the most potent CETP inhibitor currently developed and has renewed the interest in this class of agents, probably focusing on agents that reduce LDL-C rather than increasing HDL-C.

### 4.6. Small Interfering RNA

Small interfering RNAs (siRNA) are short RNA molecules that interfere with the production of different proteins through a post-transcriptional silencing. The pharmacological use of siRNA requires a “carrier” (viral or non-viral vectors) in order to deliver it directly to the intended site of action. Once in the target cell, siRNA is processed in the cytoplasm and tied to a different protein to form the RNA-induced silencing complex (RISC). The RISC complex binds to the target complementary mRNA, cleaving it, and then the RISC complex is reused to target another mRNA [68] (Figure 4).

Inclisiran is the first siRNA directed to the PCSK9 mRNA in the hepatic cells: the binding of Inclisiran with the PCSK9 mRNA promotes its degradation and consequently reduces circulating levels of PCSK9, increasing hepatic LDL-R. The drug is administered subcutaneously at a dose of 284 mg on days 1, 90, and 6-monthly thereafter because one molecule of this siRNA allows the degradation of multiple mRNA copies, hence producing a durable effect for 3–6 months. According to the pharmacodynamic characteristics, it is useful to remember that after 48 h from the administration of a single dose of 300 mg, the drug is no longer detectable in the blood, irrespective of liver function [69], but this drug is characterized by a long-lasting effect because of the stability of siRNA molecules deposited in intracellular endoplasmic compartments [70], to such an extent that a six-month administration of the drug produces adequate results.

Inclisiran has been tested in different populations in the ORION program, which included the Evaluate the Effect of Inclisiran Treatment on Low-Density Lipoprotein Cholesterol (LDL-C) in Subjects With Heterozygous Familial Hypercholesterolemia (ORION-9), which enrolled patients with clinical or genetic evidence of heterozygous familial hypercholesterolemia (HeFH) and LDL-C ≥ 100 mg/dL [71], the Inclisiran for Participants With Atherosclerotic Cardiovascular Disease and Elevated Low-density Lipoprotein Cholesterol (ORION-10), which recruited patients with ASCVD and LDL-C ≥ 70 mg/dL, and the Inclisiran for Subjects With ASCVD or ASCVD-Risk Equivalents and Elevated Low-density Lipoprotein Cholesterol (ORION-11) study, which included patients with ASCVD and LDL-C ≥ 70 mg/dL or patients with a high or very high risk of ASCVD and LDL-C ≥ 100 mg/dL [72]. Globally, the drug demonstrated a good efficacy: in a patient-level, pooled analysis of three phase III trials (ORION 9, 10, and 11) at 90 days follow-up, patients treated with 284 mg of inclisiran had a significant reduction of LDL-C of more than 50% compared to a placebo [38]. Moreover, inclisiran significantly reduced the rate of major adverse cardiac events [OR (95% CI): 0.74 (0.58–0.94)], even though these studies were not designed to evaluate clinical outcomes. Consequently, these findings await confirmation in a larger and dedicated outcomes trial of longer duration named the Randomized Trial Assessing the Effects of Inclisiran on Clinical Outcomes Among People With Cardiovascular Disease (ORION 4) trial that is enrolling more than 16,000 patients.

In terms of safety, a pooled analysis of seven trials (the ORION 1, 3, 5, 8, 9, 10, and 11 trials), including 3576 patients treated with inclisiran for up to 6 years and 1968 patients treated with a placebo, showed that an extended exposure to inclisiran does not result in an increased incidence of safety events with elevated tolerability [73]. No dosage adjustments are necessary in elderly patients or in the presence of renal insufficiency. Since the drug can be eliminated by hemodialysis treatment, the dialysis session should be avoided in the 72 h following the administration of inclisiran. No dose adjustment is required in patients with mild or moderate hepatic impairment, but, due to a lack of data, the use should be avoided in patients with severe hepatic impairment.

Inclisiran therapy may replace an anti-PCSK9 monoclonal antibody and, if so, it should be administered within 2 weeks after the last dose of a PCSK9-inhibiting monoclonal antibody in order to keep C-LDL levels consistently low. Alternatively, inclisiran can also be administered immediately after the last dose of a PCSK9-inhibiting monoclonal antibody [74].

In Europe, the use of inclisiran in clinical practice was approved by the European Medicines Agency (EMA) in 2020. According to the EMA data sheet, the use of inclisiran is indicated in adult individuals with primary hypercholesterolemia (heterozygous familial or non-familial) or mixed hypercholesterolemia in combination with a statin or statin plus another lipid-lowering agent, if they do not reach the C-LDL target despite the maximum tolerated dose of statin, or alone or in combination with other cholesterol-lowering agents in patients intolerant to statin [74].

Another siRNA aimed at controlling LDL-C levels is lepodisiran, which is directed at the synthesis of apolipoprotein (a), and it showed a significant efficacy in the reduction of Lp (a) levels. In patients without known cardiovascular disease and baseline Lp (a) levels higher than 30 mg/dL, 608 mg of lepodiseran lowered Lp (a) levels more than 90% at 48-week follow-up compared to a placebo [75].

Olpasiran is a different siRNA agent that acts by preventing the assembly of the Lp (a) in the hepatocyte, reducing its levels. In the phase 2 Olpasiran Trials of Cardiovascular Events And LipoproteiN(a) Reduction—DOSE Finding Study (OCEAN a DOSE trial), Olpasiran reduced the Lp (a) concentration by more than 95% as compared with a placebo in a dose-dependent manner when administered every 12 weeks [76].

Another siRNA acting by interfering with the Lp (a) production is under evaluation (SLN-360) and has been evaluated in a phase 1 trial [77].

### 4.7. Angiopoietin-like Proteins Inhibitors

Angiopoietin-like proteins (ANGPTL) are a group of proteins produced by the liver that act by inhibiting lipoprotein lipase and reducing triglycerides’ clearance. They exist in eight isoforms, but the principal actor in lipid metabolism is ANGPTL-3, which is secreted by the liver and catabolized by the kidney. ANGPTL-3 inhibits lipoprotein lipase (LPL), which is responsible for the hydrolysis of triglycerides content of very low-density lipoproteins and for their transformation into intermediate-density lipoproteins (or remnants) and then in LDL-C [78]. It also inhibits endothelial lipase, determining a rise in HDL-C levels [79].

Human genetic studies showed that subjects with heterozygous loss-of-function variants in the ANGPTL3 had significantly lower serum levels of triglycerides and LDL-C compared to subjects without these variants and decreased odds of ASCVD [80]. Based on these observations, several drugs have been developed to inactivate ANGPTL-3 with different mechanisms of action, including monoclonal antibodies, antisense oligonucleotides, mRNA interference (siRNA), gene editing, and vaccines. Evinacumab is a fully human monoclonal antibody against ANGPTL3 that could be administered subcutaneously (every week or every two weeks) or intravenously (every month). It has been tested in patients with familial hypercholesterolemia in the Efficacy and Safety of Evinacumab in Patients with Homozygous Familial Hypercholesterolemia (ELIPSE HoFH) Trial [81] or in patients with refractory hypercholesterolemia [42] despite optimal medical therapy (including statins, ezetimibe, and PCSK9i). In both studies, the use of Evinacumab was associated with a significant reduction of up to 50% of LDL cholesterol compared to a placebo. Unfortunately, these trials included a limited number of patients and were underpowered to test clinical outcomes. Evinacumab has been approved by the FDA and EMA in 2021 for the treatment of patients > 12 years old with homozygous familial hypercholesterolemia. However, according to a recent phase 3 trial showing good safety and efficacy also in patients from 5 to 11 years of age with a reduction of LDL-C of 48.3% from baseline to week 24 and only two treatment-related adverse events (nausea and abdominal pain) and one serious treatment emergent adverse effect of tonsillitis [82,83], the FDA approved Evinacumab also for patients >5 years old.

### 4.8. Antisense Oligonucleotides

Antisense oligonucleotides (ASO) are short, synthetic, single-stranded chains of nucleic acids that target a specific mRNA and are capable of slowing down or blocking the expression of protein synthesis [84]. The first ASO utilized in the treatment of hypercholesterolemia was mipomersen, which binds to the mRNA encoding for apo B100, reducing its production. Mipomersen, administered subcutaneously in three phase III trials conducted in patients with moderate to severe hypercholesterolemia, demonstrated its efficacy in reducing levels of apoB, LDL-C, triglycerides, and Lp (a) when compared with a placebo [85,86,87]. A cumulative post hoc analysis of these three randomized trials showed that mipomersen not only lowers levels of atherogenic lipoproteins, but may also lead to a reduction in cardiovascular events [88]. However, adverse reactions, such as injection site reactions and elevations in liver transaminase, were more frequently observed in patients treated with mipomersen compared to a placebo.

A different ASO targeting Lp (a) is pelacarsen, which reduces Lp (a) levels by blocking the mRNA of the Lp (a) gene. In a phase 2 trial enrolling 286 patients with established cardiovascular disease and Lp (a) levels of at least 60 mg/dL, pelacarsen reduced Lp (a) levels in a dose-dependent manner by up to 80% when administered subcutaneously [89]. Currently, a pelacarsen dose of 80 mg once a month is under evaluation in the Assessing the Impact of Lipoprotein (a) Lowering With Pelacarsen (TQJ230) on Major Cardiovascular Events in Patients With CVD (Lp(a) HORIZON) Trial (Clinicaltrials.gov number NCT04023552), including patients with previous myocardial infarction, previous stroke, or clinically symptomatic peripheral vascular disease.

### 4.9. Lomitapide

Lomitapide is an oral selective inhibitor of the microsomial triglyceride transfer protein, decreasing the production of triglycerides, chylomicrons, and very low-density lipoproteins. In patients with familial hypercholesterolemia, a dose of 1.0 mg per kilogram per day of Lomitapide decreased LDL-C by more than 50% [43]. However, elevated liver aminotransferase levels and hepatic steatosis remain a concern. Other adverse effects were nausea, vomiting, and diarrhea, and to date, no randomized clinical trials have evaluated the clinical outcomes in terms of cardiovascular events.

### 4.10. Gene Editing

Clustered Regularly Interspaced Short Palindromic Repeat Associated System 9 (CRISPR/Cas9) is a gene editing technique allowing the modification (removing, adding or exchanging) of DNA sequences [90]. A recent study showed the safety and efficacy of a CRISPR/Cas9-editing therapy designed to alter a single DNA base in the PCSK9 gene, in non-human primates and rodents. The therapeutic agent, called VERVE 101, was packaged in a lipid nanoparticle delivery vehicle and administered as a single intravenous infusion [44]. The drug was well tolerated, with a reduction of 69% in LDL-C up to 476 days of follow-up. Although CRISPR-based therapies are promising approaches, large clinical trials in humans are necessary to confirm their efficacy and safety.

### 4.11. Vaccines

Vaccination is an attractive therapy to treat patients with hypercholesterolemia, offering potential advantages over oral pharmacological or monoclonal antibody therapy, namely long-lasting immunity, considerably lower cost, and no need for frequent self-administration. Different vaccines have been tested to produce specific antibodies against the PCSK9 protein in order to reduce cholesterol levels and the best results have been obtained with the AT04A vaccine. AT04A is a short peptide (8–13 amino acids in length) mimicking parts of the human PCSK9 protein and is able to induce the formation of polyclonal antibodies directed to PCSK9. In a first study, transgenic mice were immunized subcutaneously with the AT04A vaccine, obtaining a reduction of total cholesterol, LDL-C, triglycerides, and biomarkers of inflammation, which was accompanied by a reduction of atherosclerotic lesions and plaques in the aortas of a mouse model of atherosclerosis [91]. Subsequently, a phase 1 randomized study conducted on 72 healthy subjects confirmed a robust, long-lasting antibody response with a significant reduction of LDL-C compared to a placebo lasting up to 70 weeks [92].

VXX-401 is another peptide-based vaccine that induces an antibody response to an epitope of the PCSK9 protein. Immunization with VXX-401 produced high-titer anti-PCSK9 antibodies in monkeys, with a concomitant reduction of 30–40% of LDL-C compared to a non-vaccinated control group [93].

All the studies performed with vaccines against PCSK9 did not show any significant adverse effects, suggesting that vaccination against PCSK9 may be a safe approach.

## 5. Conclusions

Low-density lipoprotein cholesterol (LDL-C) remains the main target of hypolipidemic treatment and statins are the first choice for therapy. These drugs are currently the heart of LDL-C lowering therapy, with widely documented effectiveness and well-known limitations. However, statin monotherapy often cannot reach the guideline-recommended goals for LDL-C reductions and is burdened by side effects. Luckily, for these cases, our pharmaceutical arsenal is currently quite satisfactory (the statin–ezetimibe–PCSK9 inhibitor combination can reduce LDL-C levels by 85%), allowing us to reach the therapeutic goal in the vast majority of patients. Moreover, the future of the management of hypercholesterolemia is bright, as many new therapies are on the horizon with the aim of a possible increase in efficacy, but mostly for a possible increase in the relapse of drug administration from daily (statins), to monthly (PCSK9i), to annually (vaccination), to finally for life (gene editing). However, more research and developmental work is still necessary before introducing these new drugs to the market.

## Data Availability

Data sharing is not applicable as no new data were created.
